# Assessing the Potential of the Plant Pellets Produced from Lignocellulosic Biomass for Seedling Growth

**DOI:** 10.3390/life16060985

**Published:** 2026-06-11

**Authors:** Kritsana Jatuwong, Worawoot Aiduang, Orlavanh Xayyavong, Tanongkiat Kiatsiriroat, Wassana Kamopas, Saisamorn Lumyong

**Affiliations:** 1 Office of Research Administration, Chiang Mai University, Chiang Mai 50200, Thailand; kritsana.ja@cmu.ac.th (K.J.); worawoot.aiduang@cmu.ac.th (W.A.); 2 Department of Biology, Faculty of Science, Chiang Mai University, Chiang Mai 50200, Thailand; xorlavanh@yahoo.com; 3 Department of Biology, Faculty of Science, Champasack University, Pakse 16010, Laos; 4 Department of Mechanical Engineering, Faculty of Engineering, Chiang Mai University, Chiang Mai 50200, Thailand; tanongkiat_k@yahoo.com; 5 Multidisciplinary Research Institute, Chiang Mai University, Chiang Mai 50200, Thailand; wassana.kamopas@cmu.ac.th; 6 Center of Excellence in Microbial Diversity and Sustainable Utilization, Chiang Mai University, Chiang Mai 50200, Thailand; 7 Academy of Science, The Royal Society of Thailand, Bangkok 10300, Thailand

**Keywords:** eco-friendly pellets, lignocellulosic biomass utilization, plant seedling growth, sustainable agriculture, SDGs 2 and 9

## Abstract

The development of sustainable and efficient plant growth substrates is crucial for modern agriculture. This study assessed the potential of plant pellets formulated from various lignocellulosic residues, either with or without bamboo biochar (BB-char) and arbuscular mycorrhizal fungi (AMF), to support seed germination and early seedling growth. Four types of residues, including coconut coir (CO), corn cob (CC), leaves from the genus *Dipterocarpus* (DL), and teak leaves (TL), were combined with soil and paper waste to produce eight pellet formulations, with commercial peat pellets serving as a control. Chemical analyses revealed significant variation among the pellet types, with pH values ranging from 6.40 to 7.65, electrical conductivity (EC) from 3.64 to 11.62 mS cm^−1^, and differences in organic matter, carbon, and nutrient contents [nitrogen (N), phosphorus (P), potassium (K)], reflecting the influence of residue type and the addition of BB-char and AMF. Phytotoxicity screening using aqueous extracts demonstrated species-specific responses, with cucumber exhibiting high tolerance across treatments, whereas chili seeds were more sensitive. Final germination percentage (FGP) and seedling growth assays in greenhouse conditions showed that pellets derived from CC and CO, particularly when combined with BB-char and AMF (T6 and T7), enhanced shoot and root development in carrot, chili, cucumber, and tomato, approaching the performance of commercial peat pellets. In contrast, DL- and TL-based pellets resulted in lower germination and growth. These findings indicate that both the physicochemical properties of lignocellulosic wastes and the combination of BB-char and AMF are important factors influencing pellet efficacy, highlighting the potential of CC- and CO-based pellets as sustainable peat alternatives for early-stage plant cultivation.

## 1. Introduction

The initial stages of plant development, particularly seed germination and seedling development, are critical factors for crop cultivation, directly influencing subsequent plant growth, yield, and overall productivity. The selection of suitable substrates plays a vital role in providing optimal physical and chemical properties such as porosity and water-holding capacity, as well as adequate nutrient availability for emerging seedlings [1,2]. Commonly, peat moss has been widely used as the primary component of nursery substrates due to its optimal physicochemical and biological properties, including high water-holding capacity and structural stability, which collectively promote optimal plant growth. Moreover, its consistent quality makes peat an ideal medium for plant growth. However, peat extraction and agricultural use raise serious environmental concerns, as peatlands are major carbon sinks, and disturbance of these ecosystems contributes significantly to greenhouse gas (GHG) emissions, thus accelerating climate change [1,3]. These concerns indicate the necessity to identify and adopt sustainable alternatives, thereby driving the search for environmentally friendly nursery substrates derived from agricultural and forestry residues. In recent years, various organic materials, such as coco peat, corn cobs, pine bark, sawdust, wood chips, and sugarcane fiber, have gained increasing attention and been used as alternative plant growth media due to their sustainability and recycling of organic wastes, as well as their potential to improve vegetable yield and quality [4,5,6,7].

Another essential factor in supporting seedling growth involves enhancing the biological and nutritional properties of plant substrates. It has been found that biochar, a carbon-rich material produced through pyrolysis of biomass, has emerged as a promising soil amendment due to its ability to improve soil structure, porosity, water retention, and microbial activity [8,9]. Similarly, AMF are involved in mutualistic symbiotic associations with plant species and are an essential component of rhizosphere microorganisms, enhancing nutrient cycling and plant nutrition [10,11]. The combined application of biochar and AMF enhances soil fertility and plant growth by increasing nutrient availability, uptake, and antioxidant activity, as well as mitigating salinity stress [12,13,14]. Additionally, biochar serves as an available carbon source for microorganisms that support AMF growth and acts as an effective carrier for AMF, promoting fungal colonization and activity through improvements in soil properties and microbial activity [14,15,16]. This synergistic effect of biochar and AMF has been shown to improve plant growth and increase yields across a variety of plants such as chili (*Capsicum flutescens* L.) [17,18], Chinese kale (*Brassica oleracea* var. *alboglabra* L.) [10], corn (*Zea mays* L.) [12,13,19], rice (*Oryza sativa* L.) [20], spinach (*Spinacia oleracea* L.) [21], and soybean (*Glycine max*) [22]. Despite these advantages, there remains limited information on the efficacy of various lignocellulosic wastes, such as agricultural and natural residues, with or without biochar and AMF additions, as peat pellet replacements for plant seedling growth.

Peat pellets, widely used as substrates in horticultural nurseries for seedling production, are primarily composed of peat moss. However, peat moss forms extremely slowly in bog ecosystems and is considered effectively non-renewable on human timescales [23]. This slow rate of formation and the environmental impacts of its harvesting raise serious ecological concerns [24]. Consequently, research attention has increased on the valorization of lignocellulosic biomass, including agricultural and natural biomass, as an alternative in horticultural growing media. According to Tumbure et al. [25], various lignocellulosic feedstocks are identified as effective and sustainable alternatives to peat, offering promising potential for use in horticultural and agricultural substrates. However, the effectiveness of these alternatives varies across different horticultural sub-sectors. Therefore, this study aimed to develop and assess the potential of pellets formulated from four types of lignocellulosic biomass, including coconut coir, corn cob, leaves from the genus *Dipterocarpus*, and teak leaves combined with and without biochar produced from bamboo (BB-char) and AMF, as alternative substrates for early seedling growth.

Although lignocellulosic wastes are abundant and have significant potential for sustainable use, their effectiveness can be limited by the presence of phytotoxic compounds. These substances can inhibit seed germination and stunt plant growth, depending on the chemical composition of the material [26]. Consequently, phytotoxicity analysis is widely used to assess the toxicity of substances before being used to prevent negative environmental impacts. Typically, phytotoxicity is assessed by evaluating the effects of substances and compounds on plant seeds, often by observing root growth responses of selected plant species [27]. Therefore, the research aimed to evaluate the chemical properties of different pellet types, the phytotoxic effects on seed germination under laboratory conditions, and the influence on seedling growth under greenhouse conditions. Experiments were conducted on five economically important vegetable crops: carrot (*Daucus carota* L.), chili (*Capsicum annuum* L.), cucumber (*Cucumis sativus* L.), holy basil (*Ocimum sanctum* L.), and tomato (*Solanum lycopersicum* L.). The overall objective was to develop alternative growing media that can be produced as low-cost and environmentally friendly, while also aiming to improve the sustainability of plant seedling growth in modern agricultural systems. This approach not only supports agricultural innovation but also enhances the value of lignocellulosic wastes through effective utilization.

## 2. Materials and Methods

### 2.1. Source of Soil, Biochar, and Arbuscular Mycorrhizal Fungi

The loam soil sample was collected from the Agricultural Innovation Research, Integration, Demonstration and Training Center (CMU Farm at Mae Hia), Chiang Mai University, Chiang Mai, Thailand. The arbuscular mycorrhiza inoculum, a commercial product (MYCOBTECH^TM^ BIO STIMULANT), was obtained from SV BIOTECH, Ratchaburi, Thailand, consisted of *Acaulospora foveata*, *Glomus etunicatum*, *G. geosporum*, and *G. mosseae*.

### 2.2. Soil and Biochar Preparation

Before use in the experiment, the collected soil was cleaned to remove wood fragments and grass residues. The cleaned soil sample were then air-dried at room temperature (approximately 25 °C) under shaded conditions until completely dry to minimize moisture variation. After that, the dried soil was gently broken down to a small size and sieved through a 3 mm mesh sieve. To produce biochar, bamboo was first dried at 80 °C for 24 h. The dried biomass was then subjected to slow pyrolysis at 500 °C for 2 h in a fixed-bed reactor [10].

### 2.3. Source of Lignocellulosic Biomass and Preparation

**Figure 1:**
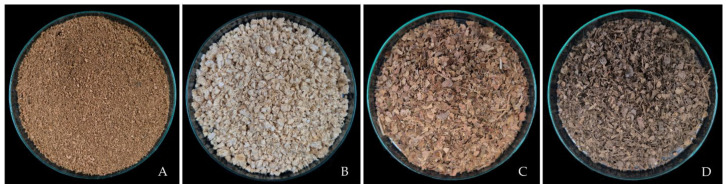
Four types of lignocellulosic biomass were used for pellet preparation. (**A**) coconut coir (CO), (**B**) corn cob (CC), (**C**) leaves from the genus *Dipterocarpus* (DL), and (**D**) teak leaves (TL).

In this experiment, commercially available horticultural-grade coconut coir (CO) dust, corn cob (CC) from agricultural residues, and leaves from the genus *Dipterocarpus* (DL) and teak leaves (TL) from natural residues were used (Figure 1). Agricultural and natural leaf residues were sourced from agricultural fields and forest areas in northern Thailand. Prior to use, the substrate was initially air-dried to reduce moisture content, then processed using a woodchipper to reduce particle size and sieved to obtain fractions with particle sizes ranging from 5 to 10 mm for CC, DL, and TL [28].

### 2.4. Experimental Design and Pellet Preparation

**Figure 2:**
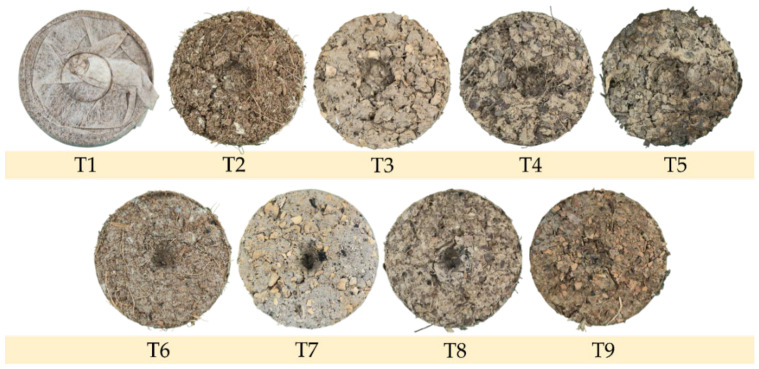
The sample of plant pellets used in this study.

The experimental design consisted of nine treatments: eight were prepared from different agricultural and natural residues, with and without biochar and AMF, and one commercially available peat pellet used as the control treatment. The preparation of plant pellets was conducted following the formulation details presented in Table 1. The soil and waste material were mixed at a 1:1 (*w*/*w*) ratio. Additionally, paper waste was added at 10%, bamboo-derived biochar (BB-char) at 5%, and AMF inoculum at 25 g per kilogram of total substrate [10]. Paper waste was used as a binding material in all formulations to enhance structural integrity [29]. After thorough mixing, the substrates were ground using a grinder and then transferred into a designed mold made from a stainless-steel tube (5.0 cm in diameter and 2.5 cm in height). The filled molds were subsequently compressed using a unidirectional cold press machine to provide consistent size and density. The obtained pellets were carefully removed and air-dried at 45 °C for 48 h to maintain their structural integrity prior to use in the experiment (Figure 2).

### 2.5. Plant Pellets Analysis

All plant pellets were analyzed for chemical characteristics. The determination of electronic conductivity (EC) and pH, a suspension was prepared at a 1:10 ratio of pellet to water. The mixture was allowed to stand for 30 min before measurement. The EC and pH were measured using an Ecoscan COND 6+ Conductivity Meter (EUTECH Instruments, Selangor, Malaysia) and a Sartorius PB-10 pH meter (Sartorius, Göttingen, Germany), respectively. The organic matter (OM) and nitrogen (N) content were determined by the Walkley and Black method [30] and the Kjeldahl method [31], respectively. For phosphorus (P) content, the vanadomolybdate colorimetric method was used [32], while potassium (K) concentration was determined by atomic absorption spectrophotometry (AAS) [33]. All chemical analyses were performed by the Department of Plant Sciences and Soil, Faculty of Agriculture, Chiang Mai University, Thailand.

### 2.6. Determination of Pellet Phytotoxicity on Seed Germination and Seedling Growth with Various Plant Species

#### 2.6.1. Plant Materials and Preparation

Phytotoxicity was assessed using five different plant species, including carrot (*Daucus carota* L.), chili (*Capsicum annuum* L.), cucumber (*Cucumis sativus* L.), holy basil (*Ocimum sanctum* L.), and tomato (*Solanum lycopersicum* L.), to evaluate the effects on seed germination and seedling growth. Each plant seed was surface-sterilized with a 0.5% sodium hypochlorite solution (*v*/*v*) for 5 min, followed by 5 washes with sterile distilled water [34]. To determine the growth of seedlings, seeds were germinated on moistened paper and incubated at 25 °C for 3 days for carrot, cucumber, and tomato, 5 days for holy basil, and 7 days for chili.

#### 2.6.2. Phytotoxicity Test of Pellet Effect on Seed Germination Under Laboratory Condition

To investigate phytotoxic effects, this study conducted a seed germination assay using aqueous extracts of the tested material. Firstly, each pellet sample was mixed with deionized water at a ratio of 1:10 (*w*/*v*), homogenized and filtered through cheesecloth and filter paper. For the germination test, 2 mL of the extract was pipetted into a 9.0 cm diameter Petri dish lined with a filter paper, while distilled water was used as the control for each seed tested. Twenty pre-sterilized seeds were placed on the germination paper in each plate, with five replicates per treatment. All plates were incubated in darkness at 25 °C for 96 h [26,35]. After incubation, the number of germinated seeds was recorded, and the radicle length of each seedling was measured. The relative germination rate (GR), relative root length (RL), and germination index (GI) were calculated using the following formulas [36]:

$$\mathrm{GR}\;=\frac{\mathrm{Number}\;\mathrm{of}\;\mathrm{germinated}\;\mathrm{seeds}\;\;\mathrm{in}\;\mathrm{test}\;\mathrm{sample}}{\mathrm{Number}\;\mathrm{of}\;\mathrm{germinated}\;\mathrm{seeds}\;\mathrm{in}\;\mathrm{water}\;}$$

$$\mathrm{RL}=\frac{\mathrm{Average}\;\mathrm{radicle}\;\mathrm{length}\;\mathrm{in}\;\mathrm{test}\;\mathrm{samples}}{\mathrm{Average}\;\mathrm{radicle}\;\mathrm{length}\;\mathrm{in}\;\mathrm{water}}$$

GI= GR × RL

#### 2.6.3. Determination of Pellet Effect on Seedling Growth Under Greenhouse Condition

In the greenhouse experiment, plant seeds that had been previously germinated under controlled laboratory conditions were transplanted into the prepared pellets. After that, the healthy germinated seeds were grown in each pellet. The pellet with each treatment were randomly arranged in a greenhouse located in the Faculty of Science, Chiang Mai University, Thailand, for 14 days. Five replicates of each treatment, each treatment, were used for growing plant seedlings in a greenhouse. The average temperature and relative humidity were contained between 25 and 30 °C and 60% and 70%, respectively. Each pellet was watered every single day.

After 14 days of planting, the number of germinated seedlings was recorded for each treatment. Subsequently, each seedling was carefully separated from the planting pellet, and the roots were gently washed under running tap water to remove residual substrate and organic matter. The seedlings were then placed in plastic containers and transported to the laboratory [10,37,38]. Final germination percentage (FGP, %) was calculated using the formula FGP = (Ni/N) × 100, where Ni is the number of germinated seeds end of the experiment and N is the total number of seeds [39]. The shoot and root length of the seedlings were measured using a vernier caliper. Each treatment was conducted in five replicates.

### 2.7. Statistical Analysis

All data were expressed as means ± standard deviation and analyzed using one-way analysis of variance (ANOVA) with IBM SPSS Statistics software (version 26.0). The significant differences (*p* ≤ 0.05) between the mean value of each treatment were considered statistically significant using Duncan’s multiple range test.

## 3. Results and Discussion

### 3.1. Chemical Properties of Plant Pellets

The chemical properties of the different formulated pellets are presented in Table 2. The results revealed variations in properties among pellets, especially influenced by the individual biomass materials used in each formulation. The influence of soil pH on plant growth has attracted considerable attention, as soil pH plays a crucial role in plant growth and development due to its impact on various key soil biological and physicochemical processes such as the mineralization of organic matter, the activity of microbial enzymes, the volatilization of ammonia, and bacterial nitrification and denitrification [40,41]. In this study, the pH values ranged from 6.40 to 7.65, indicating that all treatments were near neutral to slightly alkaline. This finding is consistent with a previous study, which reported that soil pH values between 6.5 and 7.5 are generally considered favorable for the growth of many agricultural crops [41]. The EC values varied considerably, from 4.91 to 11.62 mS cm^−1^, suggesting substantial differences in soluble salt content among treatments. Notably, the commercial pellet (T1) exhibited the highest EC value (11.62 mS cm^−1^), whereas the *Dipterocarpus* leaf-based pellet (T4) demonstrated the lowest EC (3.64 mS cm^−1^). In comparison to T1, pellets formulated from lignocellulosic biomass and their mixtures with BB-char and AMF showed intermediate EC values ranging from 3.64 to 5.53 mS cm^−1^. These findings indicate that EC can serve as a useful indicator of the availability of essential nutrients, which are crucial for supporting plant growth and development [42]. The content of OM and OC showed wide variation among treatments, with values ranging from 27.53% to 74.56% for OM and 15.97% to 43.24% for OC. The commercial pellet (T1) showed the highest levels of both OM and OC, whereas DL in T4 showed the lowest content. These higher OM and OC values are consistent with previous reports indicating peat is a naturally humified organic material characterized by a high organic carbon content. Uddin et al. [43] reported that the OC content of peat typically ranges from 24.2% to 69.3%. In this study, total N content of the different pellet formulations ranged between 0.19% and 0.53%. The highest N level was observed in T1 (0.53%), followed by T9 (0.43%) and T8 (0.41%), while T3 had the lowest N content, with a value of 0.19%. The P content was consistent in most treatments, ranging from approximately 2.23% to 2.25%, whereas treatments T6–T9, which contained biochar and AMF, exhibited comparatively lower P concentrations, ranging from 0.69% to 1.07%. The K content ranged between 0.22% to 1.22%, with T1 presenting the highest K concentration, consistent with its elevated overall nutrient profile. Altogether, the variations in N, P, and K levels can be attributed to differences in the raw materials used to produce pellet formulations, as each material’s nutrient composition is essentially different [44].

### 3.2. Effect of Phytotoxicity on Seed Germination

**Figure 3:**
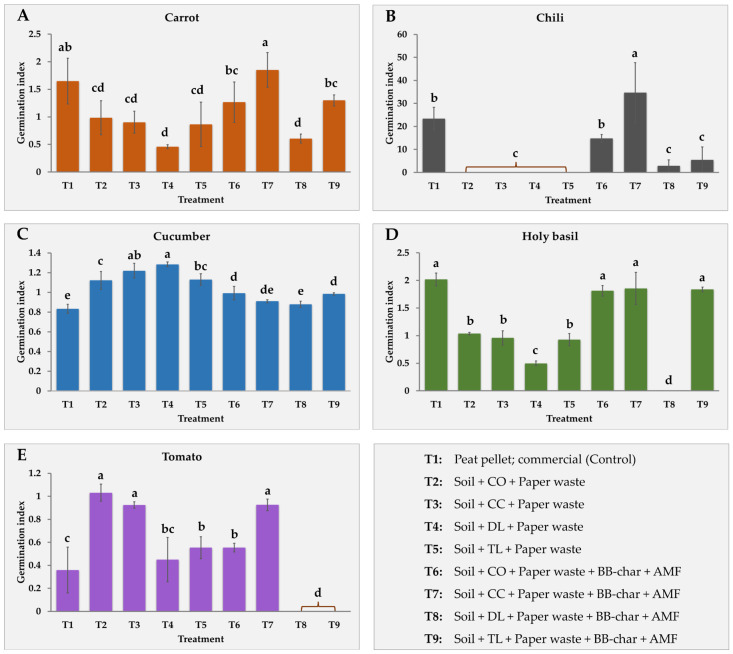
Germination index values of seeds of different plant species (**A**) carrot, (**B**) chili, (**C**) cucumber, (**D**) holy basil, and (**E**) tomato under various experimental treatments. All data are expressed as means ± standard deviation. Different letters in each treatment indicate statistical significance in Duncan’s test at *p* ≤ 0.05.

According to Zhou et al. [26], most phytotoxic compounds in plant-based residues are associated with volatile or water-soluble compounds. The results of the GI are presented in Figure 3. The GI of the five plant species varied depending on the plant pellet extracts. For carrot seeds (Figure 3A), GI values ranged from approximately 0.46 to 1.65, with the highest values observed in the CC-based treatment with BB-char and AMF (T7), which differed significantly (*p* < 0.05) from most other treatments except T1 (commercial peat pellet). In contrast, Diterocapus leave-based treatments (T4 and T8) showed the lowest GI values. In the case of chili seeds (Figure 3B), the highest GI was also observed in T7, whereas germination was completely absent, particularly T2–T5. These results indicate that chili seeds are highly sensitive to the chemical composition of the extracts. Additionally, the naturally slow and irregular germination behavior of chili seeds, as reported by Yuniati et al. [45] and Maphalaphathwa and Nciizah [46], may have influenced these results. This behavior is frequently associated with factors such as seed coat thickness, capsaicin content, variances in seed vigor, and environmental conditions, all of which can lead to uneven and lower germination rates.

In contrast to chili seeds, cucumber seeds (Figure 3C) germinated across all treatments, with GI values ranging from 0.83 to 1.28, indicating higher tolerance to the pellet extracts. This high tolerance may be attributed to the large seed size and substantial nutrient reserves, which reduce osmotic and chemical stresses [47]. The GI of holy basil seeds (Figure 3D) varied considerably among treatments, with T8 and T4 showing the lowest values, whereas T1, T6, T7 and T9 showed comparatively higher GI. Notably, plants grown on pellets derived from lignocellulosic wastes mixed with BB-char and AMF, particularly T6, T7, and T9 showed significantly higher GI values than those grown on pellets without BB-char and AMF (T2–T5). These findings align with previous studies reporting that the combination of biochar and microbial inoculants can positively influence plant growth across multiple species [8,9,10,48]. For tomato seeds (Figure 3E), GI values of plants grown on T2, T3 and T7 were significantly higher than those on most other treatments. Overall, seedlings growing on most tested pellets (T2–T7), except T8 and T9, exhibited higher GI than those grown on the commercial pellet (T1), indicating improved germination performance under these formulations.

Phytotoxic responses varied considerably among plant species, reflecting differences in physiological tolerance and sensitivity to inhibitory compounds. Such variation may also arise from differences in the chemical composition of plant residues, particularly in the type and concentration of secondary metabolites, including phenolic compounds (e.g., phenolic acids, tannins, and flavonoids), organic acids, and terpenoids. These metabolites can influence key metabolic enzymes, disrupt cellular homeostasis and membrane integrity, inhibit germination, suppress root growth, and induce oxidative stress [26,49,50]. In particular, *Tectona grandis* (teak) has been reported to contain allelochemicals that influence the germination and growth of neighboring plant species. According to the findings of Edwina and Leela [51], leaf extracts of *T. grandis* exhibited both inhibitory and stimulatory allelopathic effects on *Solanum lycopersicum* L. and *Solanum melongena* L., depending on the concentration and exposure conditions.

Several plant pellet prototypes demonstrated potential for direct seeding applications, particularly for cucumber and tomato seeds, which showed improved germination compared to commercial peat-based pellets. These findings demonstrate the importance of incorporating pre-composting or more effective stabilization processes prior to pellet production to enhance plant performance and minimize potential phytotoxic risks.

### 3.3. Effect of Different Pellets on Seed Germination

**Figure 4:**
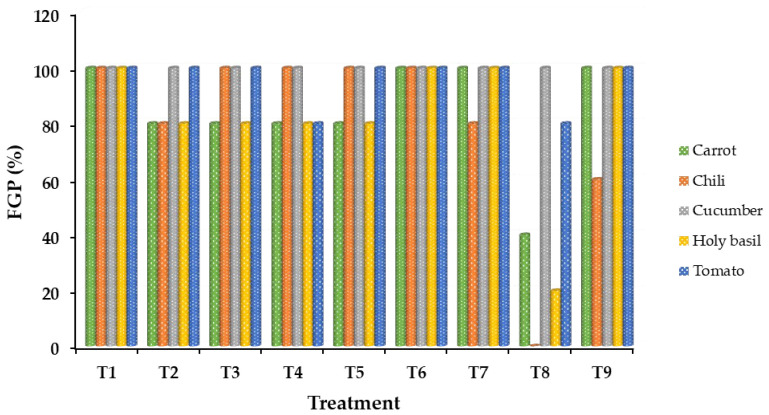
Final germination percentage (FGP) of the tested plant species grown under different pellet treatments at 14 days after sowing.

**Figure 5:**
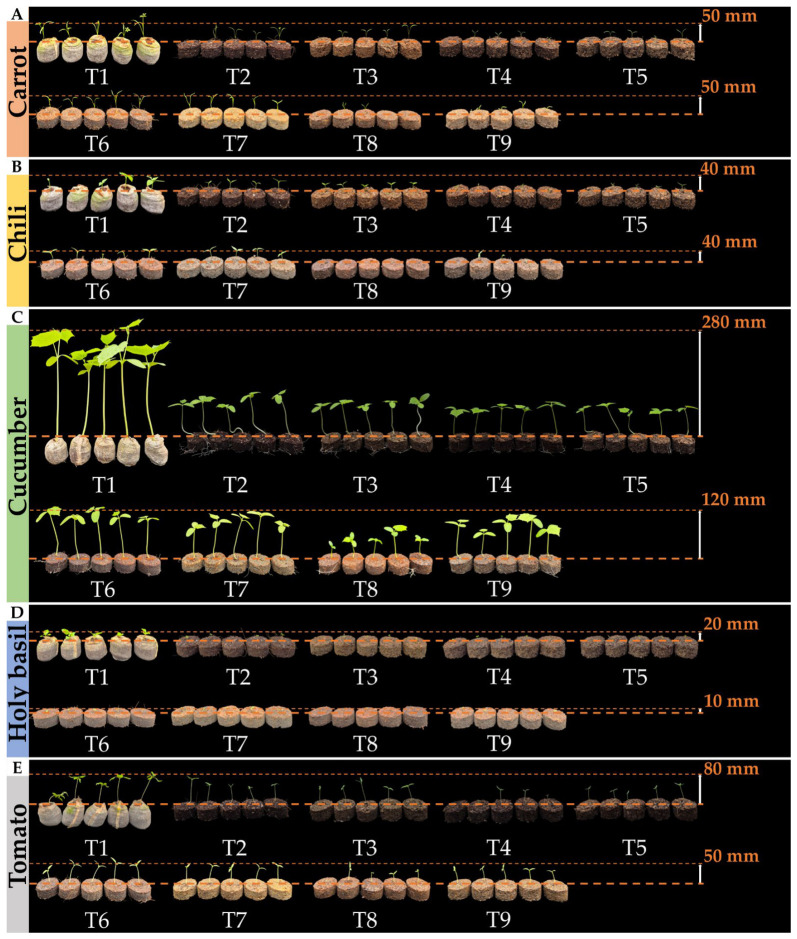
Germination of (**A**) carrot, (**B**) chili, (**C**) cucumber, (**D**) holy basil and (**E**) tomato on different plant pellets with or without bamboo biochar and AMF (for details, please see Table 2) at 14 days after growing.

The germination percentage of each plant species was initially recorded, and the final germination percentage (FGP) was calculated for the five species grown under different pellet treatments (Figure 4). Overall, FGP varied depending on both plant species and treatments, ranging from 0% to 100% germination. These results demonstrate that seed germination responses differed significantly among plant species when tested to different pellet formulations (Figure 5). Across treatments T1–T7, all plant species exhibited relatively high germination, with most FGP values ranging from 80% to 100%. Seeds grown in treatments T1 and T6 showed the highest overall germination across all species, followed by those in T7 and T9. When germination rates were compared among plant species, cucumber consistently showed high germination across all pellet formulations (Figure 5C). This observation is consistent with the laboratory plate assay, in which cucumber seeds showed the highest germination percentage among all pellet treatments. Similarly, carrot (Figure 5A) and tomato (Figure 5E) exhibited consistently high FGP values across most treatments, approaching 100%, except for carrot in T8, where the FGP decreased to 40%. In contrast, seeds of chili (Figure 5B), holy basil (Figure 5D), and carrot exhibited reduced germination in T8 compared with other treatments, indicating greater sensitivity of these species to this formulation. The particularly low FGP observed in T8 suggests that this treatment may contain factors that inhibit seed germination and early seedling development. Research on the effects of *Dipterocarpus* leaves on germination and seedling growth remains limited; however, Hossain et al. [52] reported that a mixture of forest topsoil and leaf litter of *Dipterocarpus tubinatus* enhanced the germination rate of *Leucaena leucocephala*. These contrasting findings indicate that the influence of *Dipterocarpus* residues may vary depending on several factors, such as processing methods, plant species, formulation, and chemical composition [53].

### 3.4. Effect of Different Pellets on Seedling Growth

**Figure 6:**
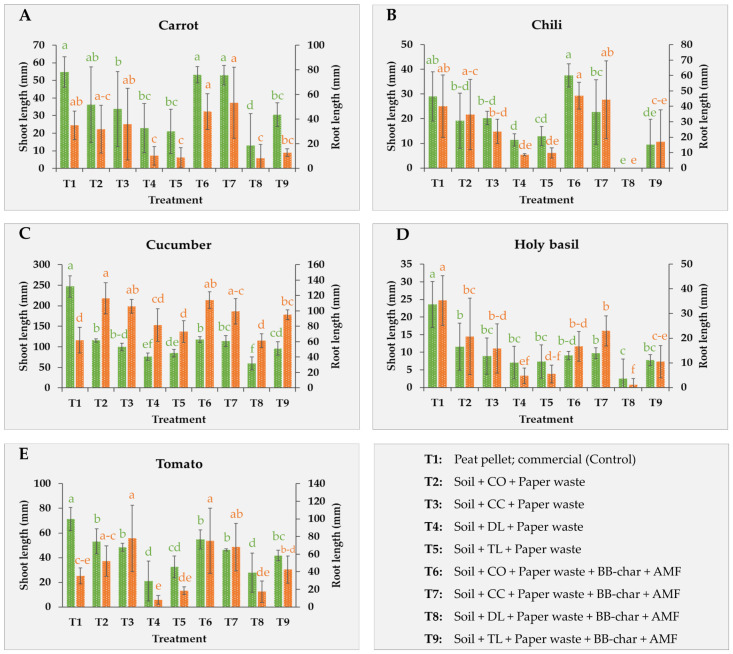
Effects of different pellet types on seedling growth, expressed as shoot and root length, in (**A**) carrot, (**B**) chili, (**C**) cucumber, (**D**) holy basil, and (**E**) tomato after 14 days of cultivation. Data are presented as means ± standard deviation. Different letters above bars indicate statistically significance between different treatments according to Duncan’s multiple range test (*p* ≤ 0.05). Green and orange bars represent shoot length and root length, respectively.

Seedlings grown on different pellet formulations exhibited significantly different growth responses across the five plant species (Figure 6). In carrot, the highest shoot length was observed in seedlings grown on T1, T6 and T7, with average values of approximately 52–54 mm, whereas seedlings grown on T8 produced the shortest shoots, averaging only 13 mm. A similar trend was observed for root length: carrot seedlings grown on T6 and T7 developed considerably longer roots (46.12 mm and 53.32 mm, respectively), while those grown on T4, T5, and T8 exhibited significantly shorter roots, ranging from 8.20 to 10.22 mm (Figure 6A). Chili seedlings also showed strong positive growth when cultivated on T1, T6, and T7, with T6 supporting the greatest shoot (37.62 mm) and root (46.90 mm) lengths. In contrast, chili seedlings grown on T4, T5, and T8 consistently exhibited the lowest shoot and root development (Figure 6B). Cucumber seedlings produced significantly longer shoots than the other species across all treatments. Seedlings grown on T1 exhibited the greatest shoot length, whereas those grown on T8 showed the shortest shoot and root development (Figure 6C). In holy basil, seedlings generally exhibited shorter shoot length across most pellet treatments, suggesting possible inhibitory effects associated with the growth medium. However, seedlings grown on the commercial pellet (T1) demonstrated the greatest shoot height. Root growth followed a pattern similar to that of shoot development, with variation among treatments (Figure 6D), indicating that root responses differed depending on the composition of the growth medium and nutrient availability [54,55]. For tomato (Figure 6E), seedlings grown on T1 showed significantly enhanced early growth performance compared with those grown on other treatments (*p* < 0.05), with the highest shoot length (71.36 mm). In contrast, seedlings grown on T4 and T8 exhibited the lowest growth responses, characterized by shorter shoots and roots. These results indicate that seedlings grown on these treatments were less effectively supported during early development.

Overall, seedling growth responses were influenced by the pellet formulations on which the plants were grown. Seedlings cultivated on the peat-based pellet (T1) consistently exhibited superior growth across all five species. This suggests that the properties of this growth medium, particularly those associated with peat moss, provided favorable conditions, including high water-holding capacity, high cation exchange capacity, adequate aeration, and low bulk density [56]. Additionally, peat-based substrates typically supply essential nutrients that support cell elongation and overall plant growth and development [57,58].

Notably, seedlings of some species responded strongly specific to alternative treatments. For example, carrot and chili seedlings cultivated on plant pellet formulations mainly comprising coconut coir and corn cob (T6 and T7) exhibited growth comparable to, or approaching, that observed in seedlings grown on the commercial pellet (T1). This result is consistent with Mahmoud et al. [6], who reported that corn cob–based substrates effectively supported plant growth. Similarly, Shuo et al. [4] reported that a substrate mixture composed of peat, vermiculite, and corn cob also presented the requirement for cucumber seedling production. However, although overall seedling response patterns were broadly similar across species, the degree of growth enhancement varied, likely reflecting species-specific differences in germination timing and early developmental stages [59]. Moreover, seedling growth responses were correlated with the chemical properties of pellets. The pH of the tested pellets ranged from 6.40 to 7.65, which is within the acceptable range for most vegetable seedlings (6.0–7.5) [60]. Despite the slightly alkaline pH of T7 (7.65), carrot seedlings responded well, indicating species-specific tolerance. Electrical conductivity (EC), which influences soluble salts and nutrient availability [61], varied widely among treatments (5.53–11.62 mS cm^−1^; Table 1). Although high EC, as observed in T1 (11.62 mS cm^−1^), can impose osmotic stress and limit water uptake, most seedlings still exhibited enhanced growth, reflecting species-specific tolerance to elevated salinity and associated compounds. The high OM (74.56%) and OC (43.24%), together with elevated levels of N, P, and K, suggest that the increased EC may be primarily attributable to a nutrient-rich organic substrate rather than to harmful salinity. In addition, organic matter is known to increase EC due to its content of soluble nutrients and salts. However, it also plays a key role in improving substrate fertility, nutrient availability, and overall plant growth [62]. Consequently, the relatively high EC did not negatively affect seed germination or seedling development in this study.

Conversely, seedlings grown on treatments with lower EC (T4, T5, T8, and T9) showed reduced growth, indicating insufficient nutritional availability for early development. Organic matter (OM) and organic carbon (OC) also played important roles by enhancing water retention, cation exchange capacity, and microbial activity, supporting root development [63]. Seedlings grown on T1, which contained the highest OM and OC levels, developed the longest shoots and roots, whereas those grown on T4, with lower OM/OC, exhibited limited growth, particularly in chili and holy basil.

Macronutrient availability also influenced seedling performance. Adequate nitrogen in T1 supported shoot development, while higher potassium levels (1.22% in T1 vs. 0.24% in T8) likely enhanced root growth and overall seedling vigor. Phosphorus concentrations were relatively similar across treatments (~0.69–2.25%). In contrast, seedlings grown on T8 and T9, which contained lower potassium levels, showed reduced development, highlighting the importance of balanced nutrient supply during early plant growth.

Seedlings cultivated on pellets formulated from CC and CO, both with and without the addition of BB-char and AMF, consistently supported stronger growth than those produced from DL and TL residues. These results are consistent with previous studies revealing that lignocellulosic sources differ substantially in their physicochemical properties, which in turn affect substrate aeration, water-holding capacity, and nutrient availability [6,52,64,65]. Additionally, the incorporation of soil amendments such as biochar and AMF has been widely reported and shown to improve soil nutrient content, cation exchange capacity, nutrient availability, microbial activity, and antioxidant enzyme activity, thereby enhancing plant growth [10,11].

## 4. Conclusions

This study demonstrated that plant pellet formulations derived from various lignocellulosic residues can serve as effective substrates for seed germination and early seedling development, particularly when supplemented with biochar and arbuscular mycorrhizal fungi. The results highlight that substrate performance is strongly dependent on the type of lignocellulosic material used. However, when combined with teak leaves and leaves from the genus *Dipterocarpus*, these additions may have a detrimental effect. Among the tested pellets, those formulated from corn cob and coconut coir, particularly when combined with bamboo-derived biochar and arbuscular mycorrhizal fungi, effectively supported seed germination and early seedling growth across various species. In comparison, commercial peat-based pellets (T1) showed the highest performance. However, the findings indicate that pellets formulated from corn cob and coconut coir combined with bamboo-derived biochar and arbuscular mycorrhizal fungi (T6 and T7) showed comparable results, and may therefore provide a low-cost alternative to peat-based substrates for early-stage plant growth. Furthermore, the pellets provide an environmentally sustainable option by utilizing agricultural residues while maintaining effective seedling performance. Overall, this study highlights that the selection, as well as the addition of biochar and microbial inoculants, are key factors in improving pellet suitability for early plant growth, offering a promising approach for valorizing agricultural residues into functional growing substrates, contributing to reduced reliance on peat and supporting more sustainable seedling production. However, T6 and T7 applications could be alternatives to commercial peat pellets, further economic evaluation under large-scale production systems is required to confirm their practical feasibility. Future studies should focus on improving pre-treatment or chemical stabilization, particularly for materials such as teak leaves and leaves from the genus *Dipterocarpus*, to reduce phytotoxic effects. In addition, further economic evaluation under large-scale production systems is required to confirm the practical feasibility of T6 and T7 as alternatives to commercial peat pellets.

## Figures and Tables

**Table 1:** Description of experimental treatments.

Treatment Number	Treatment Detail	Composition of Pellet
T1	Peat pellet (Control)	Commercial
T2	Soil + CO + PW	100 g soil + 100 g CO + 20 g PW
T3	Soil + CC + PW	100 g soil + 100 g CC + 20 g PW
T4	Soil + DL + PW	100 g soil + 100 g DL + 20 g PW
T5	Soil + TL + PW	100 g soil + 100 g TL + 20 g PW
T6	Soil + CO + PW + BB-char + AMF	100 g soil + 100 g CO + 20 g PW + 10 g BB-char + 5 g AMF
T7	Soil + CC + PW + BB-char + AMF	100 g soil + 100 g CC + 20 g PW+ 10 g BB-char + 5 g AMF
T8	Soil + DL + PW + BB-char + AMF	100 g soil + 100 g DL + 20 g PW+ 10 g BB-char + 5 g AMF
T9	Soil + TL + PW + BB-char + AMF	100 g soil + 100 g TL + 20 g PW+ 10 g BB-char + 5 g AMF

Note: CO, Coconut coir; CC, Corncob; DL, *Dipterocarpus* leaves (DL), and teak leaves (TL); AMF, arbuscular mycorrhizal fungi; PW, Paper waste.

**Table 2:** Physicochemical properties of plant pellets used in the experiment.

Treatment	pH	EC (mS/cm)	OM (%)	OC (%)	N (%)	P (%)	K (%)
T1	6.40	11.62	74.56	43.24	0.53	2.23	1.22
T2	7.51	5.53	38.41	22.28	0.29	2.23	0.41
T3	6.83	5.14	53.12	30.81	0.19	2.25	0.42
T4	6.88	3.64	27.53	15.97	0.25	2.24	0.27
T5	6.61	4.69	30.39	17.63	0.33	2.25	0.27
T6	7.48	5.51	40.45	23.46	0.29	1.07	0.38
T7	7.65	5.43	28.94	16.78	0.26	0.84	0.27
T8	6.75	4.91	41.46	24.05	0.41	0.99	0.24
T9	7.43	5.06	30.62	17.76	0.43	0.69	0.22

## Data Availability

The original contributions presented in this study are included in the article. Further inquiries can be directed to the corresponding author.
